# *‘She believed in me’*. What patients with depression value in their relationship with practitioners. A secondary analysis of multiple qualitative data sets

**DOI:** 10.1111/hex.12436

**Published:** 2016-02-18

**Authors:** John Percival, Jenny Donovan, David Kessler, Katrina Turner

**Affiliations:** ^1^ School of Social and Community MedicineUniversity of BristolBristolUK

**Keywords:** attitude, compassion, depression, practitioner–patient relationship, qualitative research, shared decision making

## Abstract

**Background:**

Clinical guidance promotes the practitioner–patient relationship as integral to good quality person‐centred care for patients with depression. However, patients can struggle to engage with practitioners and practitioners have indicated that they want more guidance on how to establish effective relationships with their patients.

**Objective:**

To identify what practitioner attributes patients with depression particularly value or find problematic.

**Method:**

A secondary analysis of data collected during four qualitative studies, all of which entailed interviewing patients diagnosed with depression about their treatment experiences. Patients in the four studies had received different treatments. These included antidepressants, cognitive behaviour therapy, facilitated physical activity and listening visits. We thematically analysed 32 patient accounts.

**Results:**

We identified two complimentary sets of important practitioner attributes: the first based on the practitioner's bearing; the second based on the practitioner's enabling role. We found that patients value practitioners who consider their individual manner, share relevant personal information, show interest and acceptance, communicate clearly and listen carefully, collaborate on manageable goals and sanction greater patient self‐care and self‐compassion. It was also evident that patients receiving different treatments value the same practitioner attributes and that when these key practitioner qualities were not evident, patients were liable not to re‐attend or comply with treatment.

**Conclusion:**

The practitioner attributes that patients with depression most value have a positive impact on their engagement with treatment. Patients emphasise the importance of a practitioner's demeanour and encouragement, rather than the amount of time or specific treatment a practitioner is able to provide.

## Introduction

It is currently estimated that between 15 and 20% of British people will suffer a depressive disorder at least once in their lives^1^ and that by 2020, depression will be the second most disabling condition worldwide.^2^ Treatments for depression in primary care typically include talking therapies, such as cognitive behavioural therapy (CBT), antidepressant medication or a combination of the two. In addition, physical exercise programmes may be offered to patients with mild to moderate levels of depression.^3^

Adherence to pharmacological, psychotherapeutic or physical activity‐based treatments is a persistent challenge for practitioners and patients alike.^4^, ^5^, ^6^, ^7^, ^8^ A significant factor influencing treatment compliance is the quality of the practitioner–patient relationship, particularly in regard to issues of openness, trust and decision sharing.^9^, ^10^, ^11^, ^12^ In respect of the GP–patient relationship, such issues may also influence the quality of communication and decision making that leads to the appropriateness or otherwise of treatment choice.^13^

The National Institute for Clinical Excellence (NICE) guidance^14^ on the management of depression refers to the central importance of providing person‐centred care based on an effective practitioner–patient relationship, with good communication an essential feature alongside a partnership approach to decision making. The importance of the practitioner–patient relationship in the area of depression management is supported by research in various primary care settings and in regard to a wide range of treatments. Studies indicate that patients with depression who have a constructive relationship with their practitioner are more likely than patients who lack such a relationship to develop trust, comply with prescribed medication or physical activity programmes, complete therapy and experience symptom reduction and improved outcomes.^15^, ^16^, ^17^, ^18^, ^19^, ^20^, ^21^

Despite clinical guidance and research highlighting the need for a positive patient–practitioner relationship, current economic circumstances and the NHS policy drive for quicker, cheaper, target‐focused health services, meaning a patient‐centred approach, may not be protected^22^, ^23^, ^24^ and practitioners may increasingly doubt its role and efficacy.^25^ An additional challenge to pursuing effective care for patients with depression is that we have limited knowledge of how patients and practitioners interact and its impact on therapeutic process.^9^, ^26^ Furthermore, the research conducted to date on patients' experiences of being treated for depression has focused on their views of specific treatments; we know very little about what elements of the practitioner–patient relationship that patients particularly value or struggle with, and whether this changes depending on patient circumstances, practitioner role or treatment type. It is important that we address these gaps in knowledge to form a more consolidated understanding of ways in which the practitioner–patient relationship can be developed within a clinical context and used to encourage patients to engage and comply with treatments.

## Methodology

This paper is based on a secondary analysis of data collected during four qualitative studies carried out between 2006 and 2011, all of which were nested within large, multicentred primary care depression trials, funded through the Health Technology Assessment (HTA) programme. Each study entailed conducting in‐depth interviews with participants diagnosed with depression and employed topic guides that included questions prompting participants to discuss their experiences of health‐care practitioners.

Treatments assessed within the trials included antidepressants, face‐to‐face CBT, online CBT, facilitated physical activity (FPA), listening visits and usual care, which was defined as treatment provided by the individual's GP. They were delivered by various practitioners who differed in terms of profession, training and specialist knowledge, that is GPs, CBT therapists, physical activity facilitators (PAFs) and research health visitors (RHVs). The PAFs and RHVs had received specific training for their role in the relevant study. Treatments delivered within the trials were aimed at different groups of patients with depression: women with postnatal depression, patients with a new episode of depression and patients with treatment resistant depression. Patients had been recruited to these studies in five UK locations: Bristol, Exeter, London, Manchester and Glasgow. Table 1 provides relevant details of the trials.

**Table 1:** The trials in which the qualitative studies were nested and details of the interviews held

Trial	Trial aim	Interventions defined	Patients interviewed
1	Evaluate clinical and cost‐effectiveness of listening visits and antidepressants as treatments for post‐natal depression	Up to 8 weekly listening visits were delivered by research health visitors (RHV) in the woman's own home.	27 trial participants; 17 had been randomized to listening visits and 10 had been allocated to antidepressants.
2	Assess the clinical and cost‐effectiveness of a facilitated physical activity (FPA) intervention plus usual care, vs. usual care alone, for patients with a new episode of depression	The physical activity intervention was delivered by physical activity facilitators (PAFs) over 6–8 months. PAFs used techniques based on motivational interviewing and behavioural strategies.	33 trial participants; 19 had been randomized to facilitated physical activity (FPA) plus usual care, the rest to usual care only. 21 of the 33 were interviewed again 9 months later.
3	Examine the clinical and cost‐effectiveness of CBT plus usual care, vs. usual care alone, for patients with treatment resistant depression	Face‐to‐Face CBT was delivered by CBT therapists. Patients were allowed up to 18 one‐hour sessions, in the patient's own GP surgery or nearby NHS or University premises.	40 trial participants interviewed; 26 had been randomized to CBT plus usual care and 14 to usual care alone.
4	Investigate the clinical and cost‐effectiveness of online CBT for patients with a new episode of depression	Online CBT was delivered by psychologists. Patients were offered up to 10 sessions.	24 patients interviewed prior to receiving online CBT. 20 of these participants were interviewed again having completed (*n* = 15) or withdrawn from treatment (*n* = 5)

We purposefully selected transcripts to ensure we had maximum variation regarding treatment allocation, participant gender and age. 35 transcripts were analysed in total. As three patients in our sample had been interviewed twice, we therefore examined data from 32 patients. Characteristics of the sample are included in Table 2.

**Table 2:** Participant characteristics and numbers (*n* = 32)

Trial (No)	Treatment allocation (No)	Age (No)	Gender (No)
1 (8)	Face‐to‐face CBT (4)	20–29 (8)	Male (12)
2 (8)	Facilitated physical activity (5)	30–39 (9)	Female (20)
3 (10)	Online CBT (6)	40–49 (7)	
4 (6)	Listening visits (5)	50–59 (2)	
	Usual care/antidepressants (12)	60–69 (6)	

We employed thematic analysis, which is an effective approach for the investigation of psychological themes as well as secondary studies of primary research.^27^, ^28^ Two of the authors (JP and KT) initially read eight transcripts several times, before separately identifying and coding emerging concepts and themes. Further transcripts were then added to the sample to ensure a sufficient spread of patient characteristics, studies and treatments. Coding frames were regularly compared and refined in terms of new codes being added and existing codes deleted or defined more clearly. Additional transcripts were coded until data saturation was reached. New emerging themes were added to the coding frame during this process. Transcripts were coded in NVivo 10 to enable researchers to electronically code and retrieve data pertaining to specific codes.

In this article, we include transcript extracts to illustrate certain points. The source of each quotation is identified using the treatment name, in abbreviated form as shown in Table 1, followed by the participant's identification number. Most of the accounts analysed for this paper focused on the interaction participants had with the individual delivering care in the trial. However, participants also talked about other practitioners, for example counsellors and practice‐based health visitors. We included these accounts in the analysis.

## Results

The presentation of our results below has been shaped by themes raised by participants and by the resonance of those themes with the work of psychologists who emphasized the dynamic role of the practitioner–patient relationship in effecting change.^29^ When detailing participants’ accounts of their relationship with practitioners, we focus on what was needed in order for participants to engage with the practitioner, comply with treatment and benefit from the interaction. According to these accounts, two complimentary sets of practitioner attributes were at work. The first set, helping provide the foundation for the relationship, was based on the practitioner's bearing (characterized by approachability, empathy, supportiveness and active listening). The second set, helping sustain and develop the relationship, was based on the practitioner's enabling role with the participant (exemplified by enhancing patient decision making and encouraging patient self‐care).

These attributes, the ways in which they were conveyed and their impact on participants, are outlined in Table 3. The order of presentation generally reflects the sequence of practitioner characteristics as experienced and/or reported by patients.

**Table 3:** Practitioner attributes, their transmission and effect

Practitioner attributes	Manner conveyed	Impact on participants
Approachability	Friendly bearing; ’Light touch’; Sharing relevant personal information	Put at ease; Feel a rapport; Willingness to engage with practitioner;
Empathy	Tune in to patient's feelings; Non‐judgemental; Praise patient progress	Improved self‐image; Raised morale; Inclined to continue with treatment;
Support	Caring attitude; Flexibility; Proactivity	Greater confidence; Cope better with pressure; Motivated to risk change
Active listening	Attentiveness; Thoughtfulness; Detailed focus	Able to express feelings; Increased clarity of thought; More positive outlook
Enhance patient decision making	Provide clear explanation; Collaborate on problem‐solving; Advise on incremental goals; Provide reassurance	Clearer understanding of treatment aims; Increased awareness of strategy options; Able to plan and pursue manageable changes
Encourage patient self‐kindness	Promote patient self‐care; Aid relinquishment of worthlessness and guilt; Sanction patient self‐determination and self‐kindness	Stronger sense of self‐worth; Attend more readily to own health needs; Safeguard sense of personal control

### Approachability

The accounts of participants from all four studies indicated that their first meeting with the practitioner established the tone and strength of their relationship. Participants valued practitioners with a ‘light touch’, who were accessible on an informal, ‘human’, level and whose receptivity created space to admit and discuss emotional difficulties:

I really got a sense that [online CBT practitioner] had a good sense of humour…that was really important for me, to have that sort of rapport, you know, somebody not very starchy…there was that slight banter. [online CBT 18]

She [RHV] wasn't condescending…So I think she made you feel comfortable…I think that's probably why I opened up so quickly…I felt at ease straight away. [LV 22]

An additional way in which practitioners conveyed approachability, and helped participants feel at ease, was by sharing relevant personal information. This helped participants to feel less socially excluded and to sense that the practitioner understood what they were saying:

She [GP] had suffered with postnatal depression and said so to me, which immediately made me feel better. [LV 1]

When participants talked of practitioners who they had experienced as unapproachable, they lamented the lack of a ‘relationship’ or ‘connection’ with them.

### Empathy and positive regard

Participants greatly appreciated evidence that the practitioner empathised with their difficulties. Practitioner empathy appeared to carry an implicit message of acceptance and understanding, which contributed to participants’ perception of a facilitative working relationship and, in turn, helped build confidence in the practitioner and the participant's own potential for change:

It was really nice for me to be able to talk to [PAF], who was really understanding where I was coming from [FPA 29]

He [online CBT practitioner] knew how I was feeling… When you're talking to someone [and] they know how you're feeling, it's easier to try and change the way you feel. [online CBT 2]

Practitioner empathy and positive regard boosted participants’ morale, and to some extent offset the negative self‐image and stigma they felt as someone with depression. In this respect, participants spoke of the benefits of having a practitioner who accentuated their qualities and potential in managing depression:

He [GP] actually said to me that he was really impressed with my progress that I had made on my own, because you need somebody to say to you, ‘you're doing alright’… when a medical person shakes you by the hand and says ‘You're doing really well’, that's the biggest boost you could ever have…I was like, I'm doing really well, I'm not crazy. [CBT 29]

When a practitioner was perceived not to appreciate the patient's range of concerns, timely help could be thwarted:

I just wish, looking back, she [health visitor] had picked up on it [depression] because I was desperately in need of help…I think it's a case of she saw just the exterior of me…she didn't pick up on any of these feelings I felt. [LV 2]

LV 2, quoted above, went on to explain that she would have sought help from her GP sooner if the health visitor had been more in tune with how she felt. A few participants talked of practitioner characteristics that were the antithesis of an empathetic approach, singling out an insensitive approach that alienated the participant and led to their ending any further contact:

I went in there, he [GP] was tapping his pen like this the whole time I was talking and I just couldn't wait to get out. I thought there is no way that I'm able to convey to him how I'm feeling… I'm not [going] to that surgery any more. [online CBT 10]

### Support

In addition to experiencing the stigma associated with mental ill‐health, participants reported lack of self‐confidence and drive. As a result, they also valued a practitioner who gave practical and emotional support:

When you get low self‐esteem and your confidence drops with depression, you shut down… And that's where [PAF] came in…I got the feeling that if I really did need someone to make a phone call and find out [about group exercise provision] for me, [PAF] would have done it for me. [FPA 13]

A proactive approach by a practitioner could provide invaluable support at a difficult time and help the participant deal with various pressures or comply with further treatment. This was the case with LV 15, who had a history of antidepressant overdose and was reluctant to register with a new GP having moved home, as she felt she would be vulnerable to the risk of further overdose if, as before, her medication use was not properly monitored. Fortunately, her RHV offered to speak to the GP about this matter, and as a result, the patient felt that sufficient groundwork had been carried out to enable her to contact the GP.

When proactive thinking did not take place, initial engagement with a practitioner could feel unproductive, or a difficult situation could quickly worsen, as was the case with FPA 13, who said her GP had ignored her warnings of an impending ‘huge problem’, with the result that deterioration of her depressed state ‘may have been avoided’.

Participants also referred to the importance of the practitioner's flexibility, for example rescheduling appointment times to accommodate a change in circumstances, or meeting in the participant's home if necessary. If flexibility was not factored in, patients under duress found this an additional obstacle to involvement with treatment:

I went through a tricky phase where I couldn't make the appointments… but [CBT practitioner] was really good and she changed the times and we managed to get through it. [CBT 1]

### Active listening

Across the studies, participants commonly stated that talking with the practitioner meant they did not feel ‘so alone’, and provided them with space to open up about their depression and give vent to rarely expressed feelings, in itself something of a relief. This included participants who communicated with their practitioner online:

You've got all these feelings going round but once you talk to someone…like he [online CBT practitioner] was very helpful, he would try and get me out of that circle. [online CBT 2]

While a minority of participants who had received online CBT found the lack of face‐to‐face contact unsatisfactory, the majority, like patients across the studies, were able to form a rapport if they felt that the practitioner carefully attended to, and reflected on, what they had to say. Indeed, a feature of the relief experienced by participants was the disarmingly simple act of being listened to attentively by an independent person with whom they could talk about painful experiences. The neutral stance of a professional provided the opportunity for less guarded talk than was possible with close family or friends, with the result that ‘you say a lot more’ and ‘you want to open up’.

A key element of the benefit derived from talking openly with a professional was the increased opportunity to gain greater perspective and understanding of experiences and associated feelings, to end the circular and inconclusive ruminating that could plague patients with depression, and to start to consider possible ‘adjustments’ that might improve outlook and well‐being. Crucially, patients needed to feel they were being carefully listened to, so that the opportunity to talk authentically not only aided their understanding but also the practitioner's:

She [RHV] really listened… I could talk properly, openly, you know, without any hidden agenda, I was truly myself… I felt like she understands me, she understands where I'm coming from. [LV 19]

Clearly, practitioners may vary according to the amount of time they can give a patient, but careful reading of the transcripts suggested that patients feel they are given sufficient time when the practitioner conveys – verbally or non‐verbally – focused attentiveness. This was suggested by patients who commented that the practitioner ‘thought about sessions’, ‘listened to everything’ and ‘really listened’. Conversely, when a practitioner appeared distracted or impatient, the time available, however long, would seem insufficient:

You had that hour but the whole time you could see the [CBT practitioner] looking at the clock…and like ten minutes before [the end] she's going, ‘right I think we need to stop there now’, and it was like, why?, you know, I wasn't asking for hours and hours. [CBT 3]

Participant CBT 3, quoted above, made the decision to cease treatment, partly on account of her dismay at the practitioner's lack of attentive, active listening. When a practitioner did not listen sufficiently, a patient could also feel that something important had been missed, as was the case with LV 19, who thought that her GP could have referred her for more specialist help if he had ‘just listened’.

In addition to the examples of how practitioners could provide a foundation for patient engagement, participants’ accounts also illustrated how practitioners could build on this platform and enable participants to develop their own capabilities, particularly in regard to decision making and in practising what we have termed ‘self‐kindness’.

### Enhance patient decision making

Participants detailed the importance of receiving information from practitioners that increased their understanding of depression and enhanced their ability to evaluate treatment advice. Such information enabled them to engage with treatment and maximize its benefits.

She [online CBT practitioner] sort of explained what she was going to do, the kind of things she was going to ask, and sort of give me a reason for why she was asking them, rather than just asking me questions and I'm thinking, why does she want to know that? [online CBT 11]

Practitioners were also seen to encourage participants’ knowledge by helping them think more deeply about themselves and their situation. This helped participants clarify and prioritize goals and strategies. Participants reported that decision making was also advanced by the prac‐titioner's collaborative approach to goal setting and strategy development. Participants very often used the word ‘we’ when talking about the decision making that had taken place, and did so in a way that suggested enthusiasm for, and active engagement with, collaborative aspects of the therapeutic process:

We [patient and PAF] reviewed [goals] and sort of used that in setting the next goals that we did. [FPA 3]

We [patient and CBT practitioner] would set sort of goals and objectives of what we wanted to talk about… it was generally a sort of like joint effort. [CBT 12]

Participants often said that they struggled to find the ‘willpower’ to achieve tasks. Collaborative work with a practitioner to review incomplete goals and make changes at a more realistic pace was seen as useful:

She [PAF] was very good at making me focus on the small steps [which] very much changed my mindset…the idea of just doing something little rather than aiming for really big straight away…and [so] I've made small changes… [and] that made a difference [FPA 3]

Participants across the studies highlighted initiatives they had taken to engage or comply with treatment, such as rearranging work hours to accommodate treatment sessions, requesting a lower or higher medication dose that reflected their progress, finding the patience and resolve to talk about personal matters face‐to‐face or online, and overcoming weariness so as to exercise. Participants indicated that when their compliance or initiative faltered, working with the practitioner helped overcome obstacles and improved decision making:

I've done that [prematurely stopped taking antidepressants] before and not been ready, whereas now…with [CBT practitioner] we'll talk about it [ending anti‐depressants], we'll think about it and [I will then] talk about it with the GP. [CBT 1]

### Encourage self‐kindness

Participant accounts suggested they judged themselves harshly, blaming themselves for the problems that had arisen in their lives and struggling to focus on their own needs. An important way in which practitioners fostered participants’ strengths, and lessened their vulnerabilities, was by encouraging them to reduce pressure on themselves, attend more readily to their own needs and practise greater self‐kindness. This could take the form of encouraging participants to address debilitating feelings of guilt:

You judge yourself quite harshly …and you want to do everything right.. And I was just getting more and more anxious and I think that just made everything else worse. So she [RHV] goes, ‘Well, just try to relax and don't put any pressure on yourself and just take it one day at a time’. And I was, like, yeah, you know, little things like that make you stop. [LV 22]

When we were talking about my sexual abuse she [CBT practitioner] turned and said, ‘You know, what he did to you was wrong’, and that was a relief in itself.. coming from someone in authority, who is on your side… I've realised that I'm very, very capable…so I should be giving myself credit. [CBT 2]

A number of accounts suggested that a lack of self‐attention was also a result of the guilt participants felt for having depression, due to the burden they perceived it placed on others. As a result, when participants agreed to pay more attention to their own needs, this was often justified as beneficial, not only to themselves, but also to others:

I was sort of bottom on my list, before,…[now] I'm trying to sort of do more that's right for me. I think if it's right for me then it's right for everybody. [FPA 3]

Participant LV 22 voiced her enjoyment of her counselling sessions with the RHV as they felt like ‘me‐time’. Indeed, participants grew to appreciate the importance of safeguarding dedicated ‘me‐time’ time if they were to succeed in helping themselves. Participant LV 1, for example, would sit in her car for the duration of her Tai Chi class if the instructor did not turn up, as she regarded that time as ‘my night’.

## Discussion and conclusion

The optimum conditions for person‐centred care emphasize the importance of the relationship, the ‘climate’, created between practitioner and patient, along with the practitioner's ‘facilitative’ role in helping patients access their own ‘resources’.^29^ In considering the ways in which practitioners establish a facilitative relationship with patients, we have identified the significance patients place on having the opportunity to engage with a practitioner who is approachable, supportive and shows empathy and attentiveness. In regard to the ability of practitioners to maximize patients' confidence in their own resources, we have highlighted the importance of encouraging patients' understanding of depression and their treatment, sharing the development of decision‐making strategies, and helping patients foster self‐kindness.

### Establishing a facilitative relationship

Studies have shown that patients with mental health problems benefit from the formation of an open relationship with a practitioner, in which patients are listened to, understood and encouraged, and in which they feel safe, cared for and supported.^30^, ^31^, ^32^, ^33^ These aspects have been researched and promoted for decades in respect of psychotherapeutic treatments for patients with depression,^34^ and yet, those responsible for training one relevant professional group, CBT therapists, concede that not all practitioners feel equipped or confident to form and sustain a working relationship with their patients and welcome the opportunity to learn skills and strategies to connect more effectively.^19^

Participant accounts illustrate that practitioners’ engagement in a relationship with patients with depression is a matter of attitude and bearing, a way of being as much as doing.^35^ One feature of the practitioner's approachability, which we refer to, is the sharing of personal information by the practitioner. This is a good example of what Rogers^29^ defines as ‘congruence’, where the therapist draws on their own experience, including self‐disclosure, to assist the relationship. Congruence is perhaps what Berger^36^ had in mind when describing the importance of the country doctor's comparability to, and ‘fraternity’ with, the patient. Congruence is also pertinent to Heath's argument, based on her primary care experience, that GPs, with busy schedules and a possible tendency for ‘unthinking doing’, make better use of limited consultation time by ‘listening and noticing [and] bearing witness [through] companionship and solidarity’.^31^

The ability to discuss personal history with a practitioner in a way that feels shared can increase understanding and be part of the healing process^22^, ^37^ but studies highlight how patients with depression may find talking to a practitioner difficult, for reasons that include previous poor experience of help‐seeking, fear of being stigmatized and reluctance to waste practitioners’ valuable time.^38^, ^39^, ^40^ Active listening by the practitioner is a hallmark of good quality communication and can encourage patients to talk about their feelings. Attentiveness and active listening express a practitioner's willingness and ability to help the patient with depression^31^, ^41^ and highlight how it is the quality of time spent with depressed patients, rather than its length, that is important.^42^

### Enabling progress of patient resources

Agreement between practitioner and patient on clearly explained treatment goals and tasks enables and encourages patients to participate in shared decision making, and also strengthens the practitioner's relationship with depressed patients.^9^, ^16^, ^17^, ^38^ Shared decision making helps practitioners build camaraderie and trust with patients,^43^ increase patients' sense of control with regard to treatment discussions^44^ and motivates patients to make productive changes in behaviour.^33^

It is also important to recognize the experiences and motivation that patients bring to the interaction.^45^, ^46^ Patients' own resources can be overshadowed by rigidity in the therapeutic approach,^47^ and it is important to maximize patients' sense of control within the relationship, as this tends to be fragile for those with mental health difficulties.^44^, ^48^ The facilitative practitioner may harness patients' positive efforts and at the same time ease their distress by encouraging greater, potentially restorative, self‐kindness.

Compassion towards oneself is an important characteristic of Maslow's model of self‐acceptance and an aide to combating the root causes of psychological illness.^49^ Indeed, Gilbert^50^ argues that cognitive change does not necessarily improve the mental health of those patients with deep‐seated feelings of shame or worthlessness, who may benefit more from help to cultivate inner compassion. Self‐compassion has been defined as being kind and understanding to oneself, accepting failures and imperfections as intrinsic to the human condition, and holding a balanced view on painful thoughts, neither avoiding nor overidentifying with them.^51^ Self‐compassion programmes of various kinds, including those using randomized control trials, have produced positive results for patients with depression.^51^, ^52^, ^53^ Studies have indicated that greater self‐compassion can provide sufficient emotional security and motivation to rectify debilitating thoughts, feelings and behaviour, and help with depression.^54^, ^55^, ^56^ We have seen how patients across our four studies valued efforts to help them practice greater self‐kindness but generally patients with depression find self‐kindness hard to put into practice^57^ Practitioners may therefore usefully reinforce the importance of this focus within psychological therapies.^55^

Building and developing a relationship with patients that achieves the outcomes identified in this paper may seem challenging for busy practitioners. However, as already noted, availability of time is not the critical factor and involving patients in decision making need not take doctors more time.^58^ Important in the eyes of patients, and the main practical implication of our analysis, is an approach in which the practitioner considers their personal demeanour, may share relevant personal information, shows interest and acceptance, communicates clearly and listens carefully, collaborates on manageable goals and sanctions greater patient self‐care and self‐compassion.

A possible limitation of this study is our ‘distance’ from the primary research and its context, a concern often noted in regard to the analytic validity of secondary analysis.^59^, ^60^ In our case, this pitfall was significantly mitigated by involvement in the secondary analysis of researchers involved in the primary studies (KT, DK), whose knowledge and experience of original data helped substantiate interpretations put on it by others and thereby provided a method of corroboration, useful in secondary analysis.^61^ Another potential limitation is that the primary research did not routinely ask patients about their relationship with practitioners in the same way, or with equal emphasis, across the four studies, and so the original data vary in respect of the detail and coverage of the issue. However, our focus on the practitioner–patient relationship originated through identifying this issue as an important one in all four studies, with sufficient data in each regarding interaction between different practitioners and patients. This ‘fit’ between data and research focus, together with the richness and narrative content of the original data, is key to ensuring validity in secondary analysis.^62^, ^63^ Indeed, we have been able to highlight overlap between four patients groups in terms of significant commonalities relevant to the practitioner–patient relationship. A major strength of our study is its relevancy and reach across four data sets, given that it encompassed diverse groups of patients with depression, receiving different treatments, delivered by practitioners with varied experience, training and patient contact‐time.

In conclusion, secondary analysis of four qualitative data sets has shown how an effective practitioner–patient relationship can be developed and positively impact upon engagement with the practitioner and treatment, as well as the patient's view of themselves and their ability (emotionally, cognitively and practically) to manage their depression. This is important because in the present context of clinical governance and guidance, the quality of the practitioner–patient relationship and the safeguarding of person‐centred care assume high significance.^25^, ^35^, ^64^, ^65^ We have attended to these imperatives by identifying ways in which health practitioners can establish a supportive and enabling relationship with patients with depression.

## Source of funding

Our study was funded by the National Institute for Health Research School for Primary Care Research. The views expressed in the article are those of the authors and not necessarily those of the NHS, the NHR or the Department of Health.
